# Development and validation of a two glycolysis-related LncRNAs prognostic signature for glioma and in vitro analyses

**DOI:** 10.1186/s13008-023-00092-9

**Published:** 2023-06-24

**Authors:** Xiaoping Xu, Shijun Zhou, Yuchuan Tao, Zhenglan Zhong, Yongxiang Shao, Yong Yi

**Affiliations:** 1 Department of Neurosurgery, The Second People’s Hospital of Yibin, Yibin, 644000 Sichuan Province China; 2 Department of Health Examination, The Second People’s Hospital of Yibin, Yibin, 644000 Sichuan Province China

**Keywords:** Long noncoding RNAs (lncRNAs), Glioma, Glycolysis, Prognostic signature, Immune landscape, Immune checkpoint inhibitor (ICI)

## Abstract

**Background:**

Mounting evidence suggests that there is a complex regulatory relationship between long non-coding RNAs (lncRNAs) and the glycolytic process during glioma development. This study aimed to investigate the prognostic role of glycolysis-related lncRNAs in glioma and their impact on the tumor microenvironment.

**Methods:**

This study utilized glioma transcriptome data from public databases to construct, evaluate, and validate a prognostic signature based on differentially expressed (DE)-glycolysis-associated lncRNAs through consensus clustering, DE-lncRNA analysis, Cox regression analysis, and receiver operating characteristic (ROC) curves. The clusterProfiler package was applied to reveal the potential functions of the risk score-related differentially expressed genes (DEGs). ESTIMATE and Gene Set Enrichment Analysis (GSEA) were utilized to evaluate the relationship between prognostic signature and the immune landscape of gliomas. Furthermore, the sensitivity of patients to immune checkpoint inhibitor (ICI) treatment based on the prognostic feature was predicted with the assistance of the Tumor Immune Dysfunction and Exclusion (TIDE) algorithm. Finally, qRT-PCR was used to verify the difference in the expression of the lncRNAs in glioma cells and normal cell.

**Results:**

By consensus clustering based on glycolytic gene expression profiles, glioma patients were divided into two clusters with significantly different overall survival (OS), from which 2 DE-lncRNAs, *AL390755.1* and *FLJ16779*, were obtained. Subsequently, Cox regression analysis demonstrated that all of these lncRNAs were associated with OS in glioma patients and constructed a prognostic signature with a robust prognostic predictive efficacy. Functional enrichment analysis revealed that DEGs associated with risk scores were involved in immune responses, neurons, neurotransmitters, synapses and other terms. Immune landscape analysis suggested an extreme enrichment of immune cells in the high-risk group. Moreover, patients in the low-risk group were likely to benefit more from ICI treatment. qRT-PCR results showed that the expression of *AL390755.1* and *FLJ16779* was significantly different in glioma and normal cells.

**Conclusion:**

We constructed a novel prognostic signature for glioma patients based on glycolysis-related lncRNAs. Besides, this project had provided a theoretical basis for the exploration of new ICI therapeutic targets for glioma patients.

**Supplementary Information:**

The online version contains supplementary material available at 10.1186/s13008-023-00092-9.

## Introduction

Gliomas make up virtually 80% of all lethal primary brain tumors, which seriously threatening human health and causing a heavy burden to the social economy [18]. Despite many advances in deciphering the underlying molecular mechanisms of gliomas, the efficacy of clinical comprehensive treatment options have reached a bottleneck, and as a result, the long‐term survival rate of glioma patients remains poor. The complex heterogeneous of tumor and the unique microenvironment of the brain present major challenges in treating gliomas. Therefore, there remains an urgent need to comprehensively understand the tumor microenvironment and identify a valuable biomarker for predicting the prognosis of glioma patients, which may lead to the development of new potential therapeutics for glioma patients.

Abnormal metabolism and immune evasion are two hallmarks of cancer [20]. It has been under extensive exploration in the hope of discovering new targets and effective therapies. Like other cancers, glioma presents a unique metabolic state known as the Warburg effect [21]. The exact mechanism of metabolic transformation remains unclear, previous studies have showed that the metabolic mode can switch during tumor progression. Reversal of the Warburg effect could potentially serve as a novel therapy for glioma [21, 24]. Recently, studies have identified alterations in tumor metabolism can also contribute to a potent tolerogenic immune environment [15]. With continued advancement in both of these research disciplines, the relationship between tumor metabolism and their subsequent influence on immune regulation has become increasingly recognized as an important factor contributing to tumor growth and progression. A recently study showed that there is a close correlation between the altered metabolic landscape and increased activity of infiltrated immune cells within the tumor microenvironment [17, 27]. Furthermore, CTLA-4 blockade has been found to promotes metabolic fitness and the infiltration of immune cells, especially in glycolysis-low tumours [31]. These findings suggest that the complex interdependencies exist between tumor metabolic and immune responses.

LncRNAs have been reported to be dysregulated in various types of cancers, it can regulate cancer cell proliferation, invasion, metastasis, and therapeutic resistance [11]. Tumor glycolysis could be used as a potential therapeutic target for cancer [8], the major challenge lies in the fact that metabolism is a universal cellular process [23]. Accumulating evidence has shown that lncRNAs can alter glucose metabolism either directly or indirectly [16, 29, 30, 33]. Therefore, lncRNAs are considered as a promising strategy for addressing this challenge [2, 7, 13].The relationship between glycolysis-related lncRNAs and glioma prognosis has rarely been studied. A recent study identified six glycolysis-related lncRNAs significantly related to prognosis of glioma patients through analyzed CGCA database [25, 26]. However, comprehensive analysis focusing on glycolysis-related lncRNAs exerts on glioma patients in TCGA, as well as whether these glycolysis-related lncRNAs have potential impact on immune or not is lacking. In the present study, we implemented studies with transcriptome and clinical data of glioma from The Cancer Genome Atlas (TCGA) projects to explore the glycolysis-related lncRNAs and immunity in glioma, to identify the glycolysis-related lncRNAs and relationship between the tumor glycolysis and immune cells infiltration on glioma patients.

## Results

### Consistent clustering of glioma and the survival rate of clusters

**Fig. 1:**
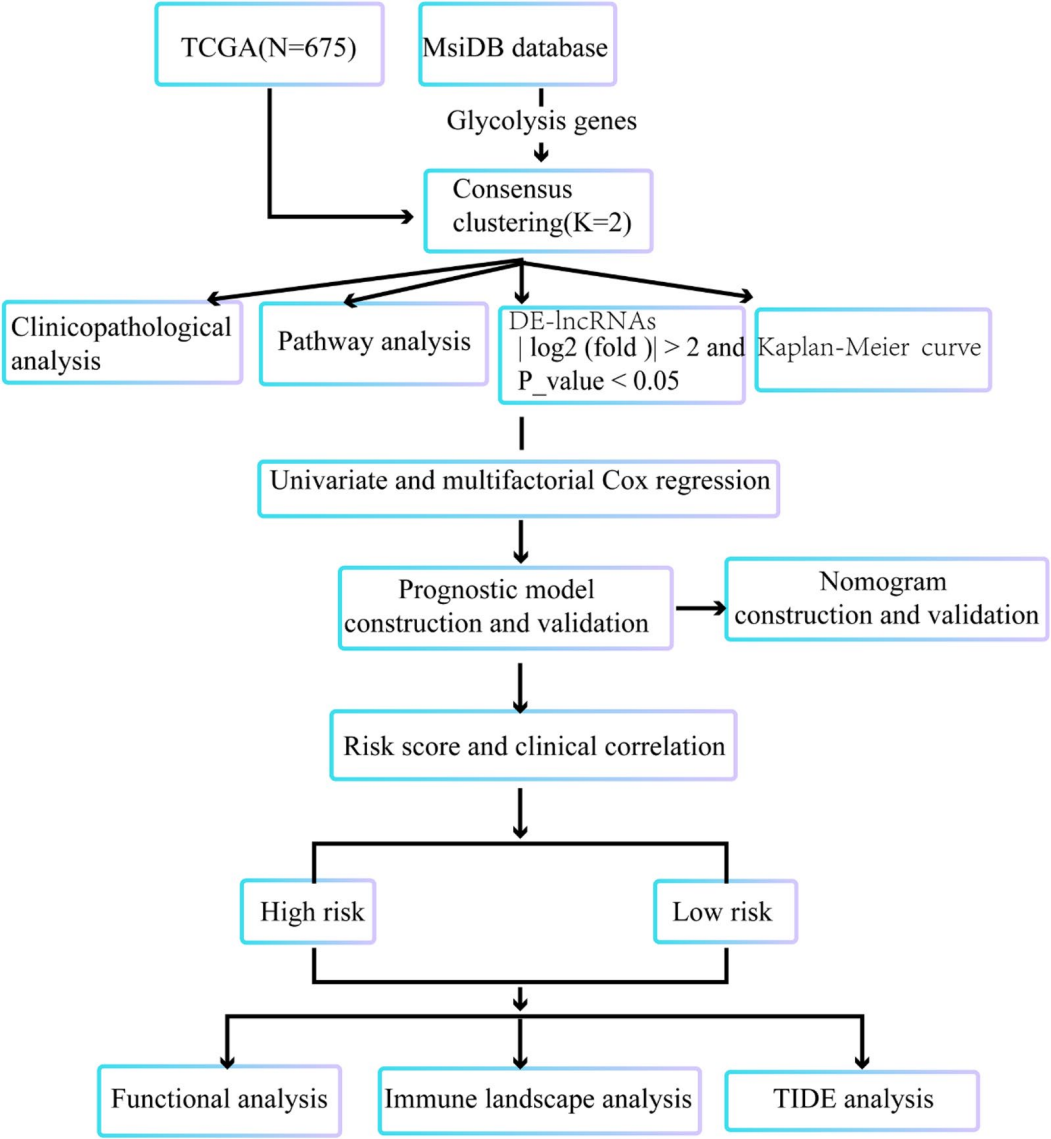
Flow chart of the study design

**Fig. 2:**
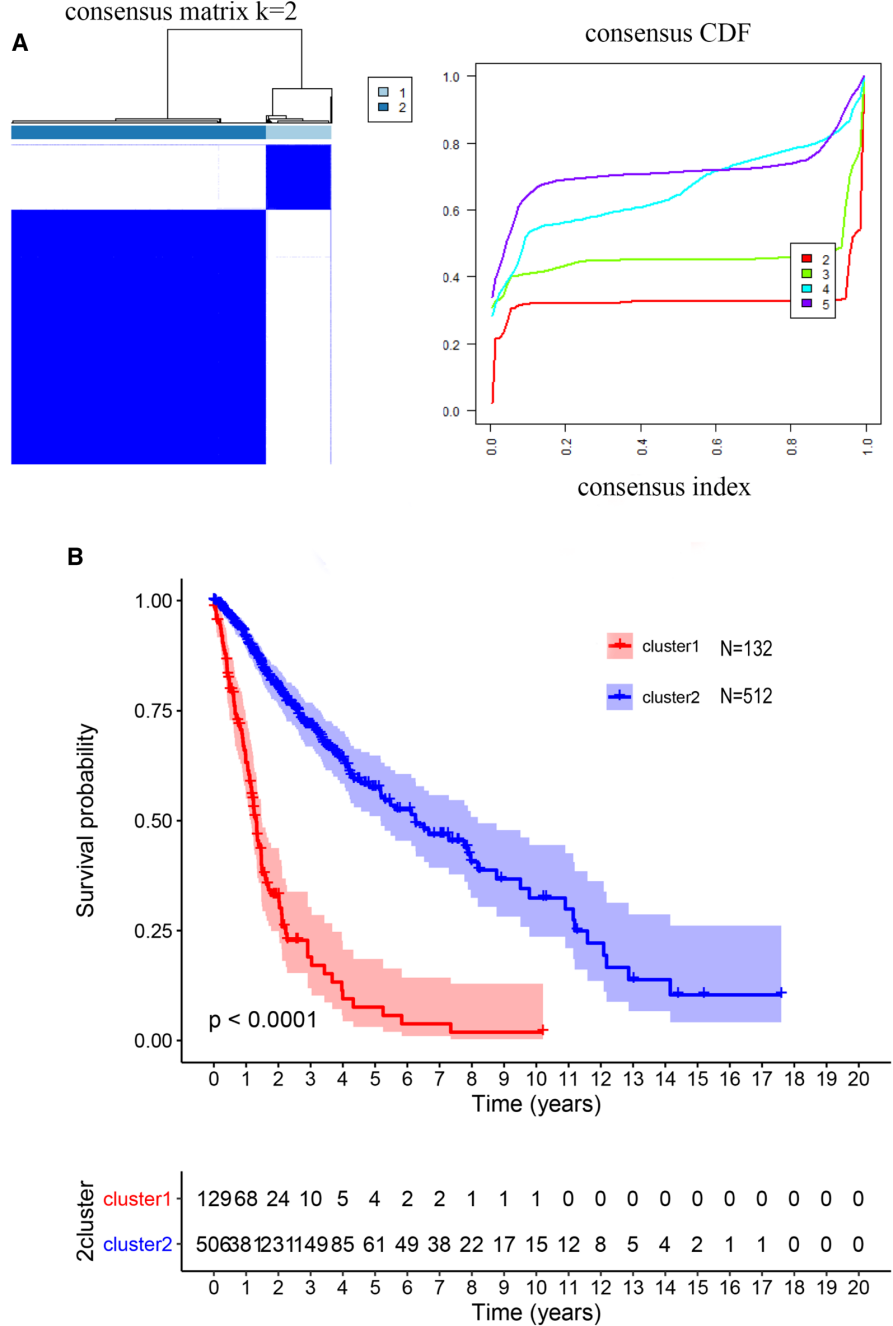
**A** Identification of consistent clustering by glycolysis genes in the TCGA database. **B** The “Kaplan–Meier”overall survival (OS) curve of two clusters defined by consistent expression

The study was conducted as described in the flow chart (Fig. 1). Consistent clustering analysis of glioma patients was performed based on the expression of glycolysis genes in the TCGA database. Finally, patients were decided to be divided into 2 sample clusters, with cluster 1 containing 132 samples and cluster 2 containing 512 samples (Fig. 2A). The K-M suggested a significant difference in cumulative mortality at 20 years between the two subtypes (*P* < 0.0001; Fig. 2B). In the glioma cohort, the best outcome was found in patients classified as cluster 2. The next question was to determine the relationship between sample clustering results and clinicopathological data (treatment, age, gender, IDH, grade, and MGMT). Matching these results to all clinicopathological characteristics, significant correlations had been noted among treatment, age, IDH, grade, MGMT and the two clusters (Table 1). In summary, these results appeared to imply that glioma could be stratified according to the expression pattern of glycolysis genes.

**Table 1:** The relationship between sample clusters and clinical characteristics of glioma patients

		Cluster1	Cluster2	P
Treatment	no PRT	16	108	3.04E−05
	RT	7	67	
	PT	6	48	
	PT + RT	95	247	
Age	> 60	56	80	7.33E−11
	< 60	68	390	
Gender	male	78	262	0.1831
	female	46	208	
IDH	IDH-mut	15	321	< 2.2e−16
	IDH-wt	78	89	
Grade	G2	4	186	< 2.2e−16
	G3	19	195	
	G4	70	29	
MGMT	Methylated	43	334	3.76E−12
	Unmethylated	50	76	

*no PRT* no Pharmacotherapy and radiotherapy; *PT* Pharmacotherapy; *RT* radiotherapy

Furthermore, we also assessed differences in pathway enrichment scores between the two clusters based on the KEGG pathway using the ssGSEA algorithm (Additional file 6: Table S6). Unexpectedly, glycolytic (‘Glycolysis/Gluconeogenesis’) and glycan synthesis and metabolic (‘Amino sugar and nucleotide sugar metabolism’, ‘Glycosaminoglycan biosynthesis-keratan sulfate’, ‘Mannose type O-glycan biosynthesis’, ‘Galactose metabolism’, etc.) pathways were significantly different between the two clusters. Also, immune response- (‘cAMP signaling pathway’, ‘IL-17 signaling pathway’, ‘Cell adhesion molecules’, ‘Intestinal immune network for IgA production’, etc.) and immune cell (‘Th17 cell differentiation’, ‘Th1 and Th2 cell differentiation’, etc.)-related pathways were notable between Cluster 1 and Cluster 2. Further, PD-L1 expression and PD-1 checkpoint pathway in cancer also showed substantial variations.

### Establishment of a prognostic signature based on the DE-glycolysis-related lncRNAs

**Fig. 3:**
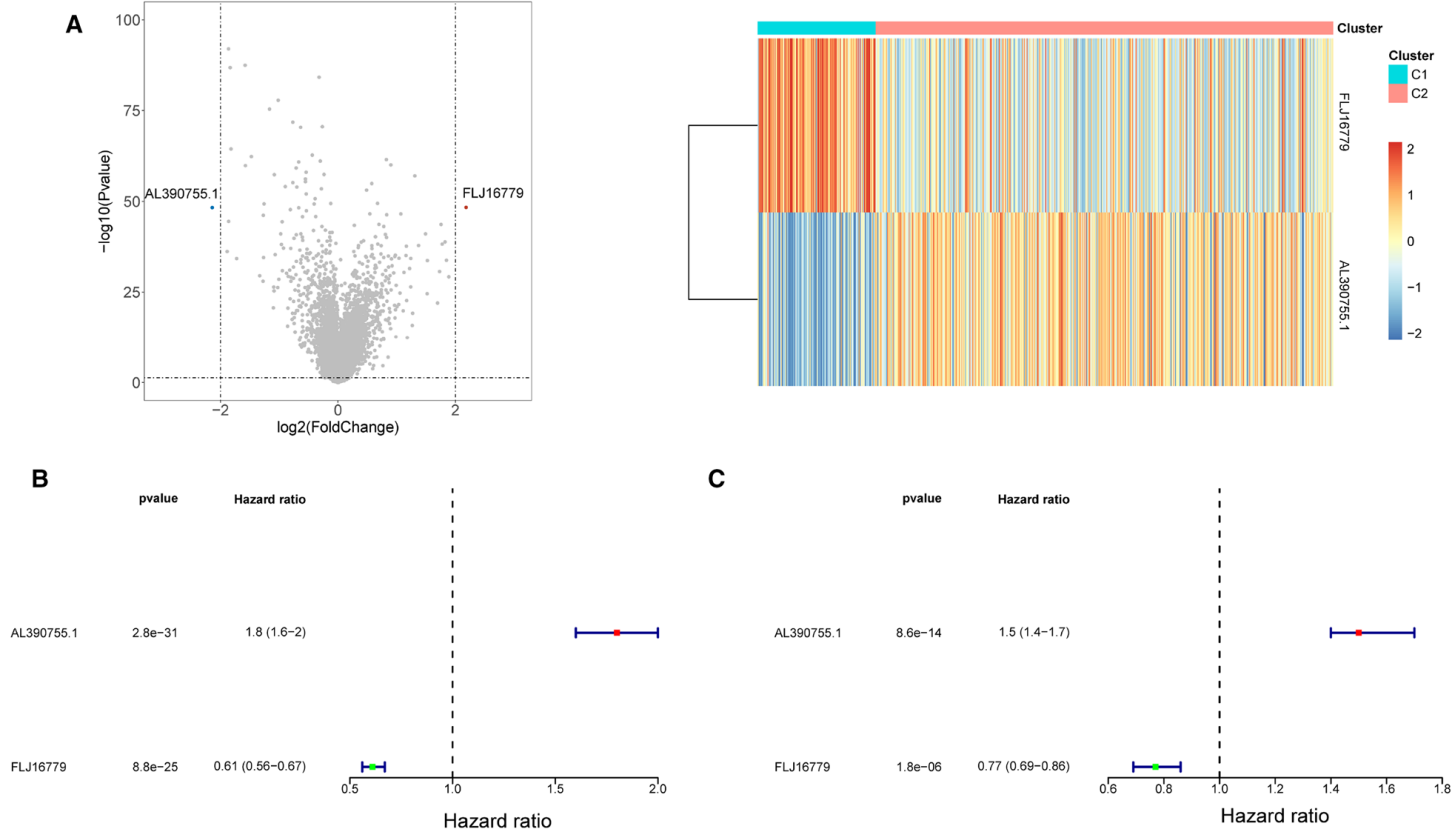
**A** Volcano plot and heatmap of differentially glycolysis-related lncRNAs in both cluster 1 and cluster 2 subtypes. **B** Univariate cox regression analysis in the training set. **C** Multifactorial cox regression analysis in the training set

We identified a total of 2 DE-lncRNAs from both subtypes, relative to cluster 2, lncRNA *FLJ16779* was the up-regulated gene and lncRNA *AL390755.1* was the down-regulated gene (Fig. 3A; Additional file 7: Table S7). These genes were defined as DE-glycolysis-related lncRNAs for the subsequent analysis. To verify whether these 2 DE-lncRNAs were the risk factors for glioma, we performed a univariate Cox regression analysis in the training set. Coincidentally, all DE-lncRNAs met *P* < 0.05 (Fig. 3B). Subsequently, a multifactorial Cox regression analysis with a STEP function indicated these 2 lncRNAs as the optimal variables for constructing a prognostic signature (Fig. 3C). Specifically, *AL390755.1* (*P* = 8.59E−14) was possible a risk factor for glioma (HR = 1.38), whereas *FLJ16779* (*P* = 1.82E−6) was inferred to be the oncogene (HR = 0.77).

### Assessment and validation of the effectiveness of the prognostic feature

**Fig. 4:**
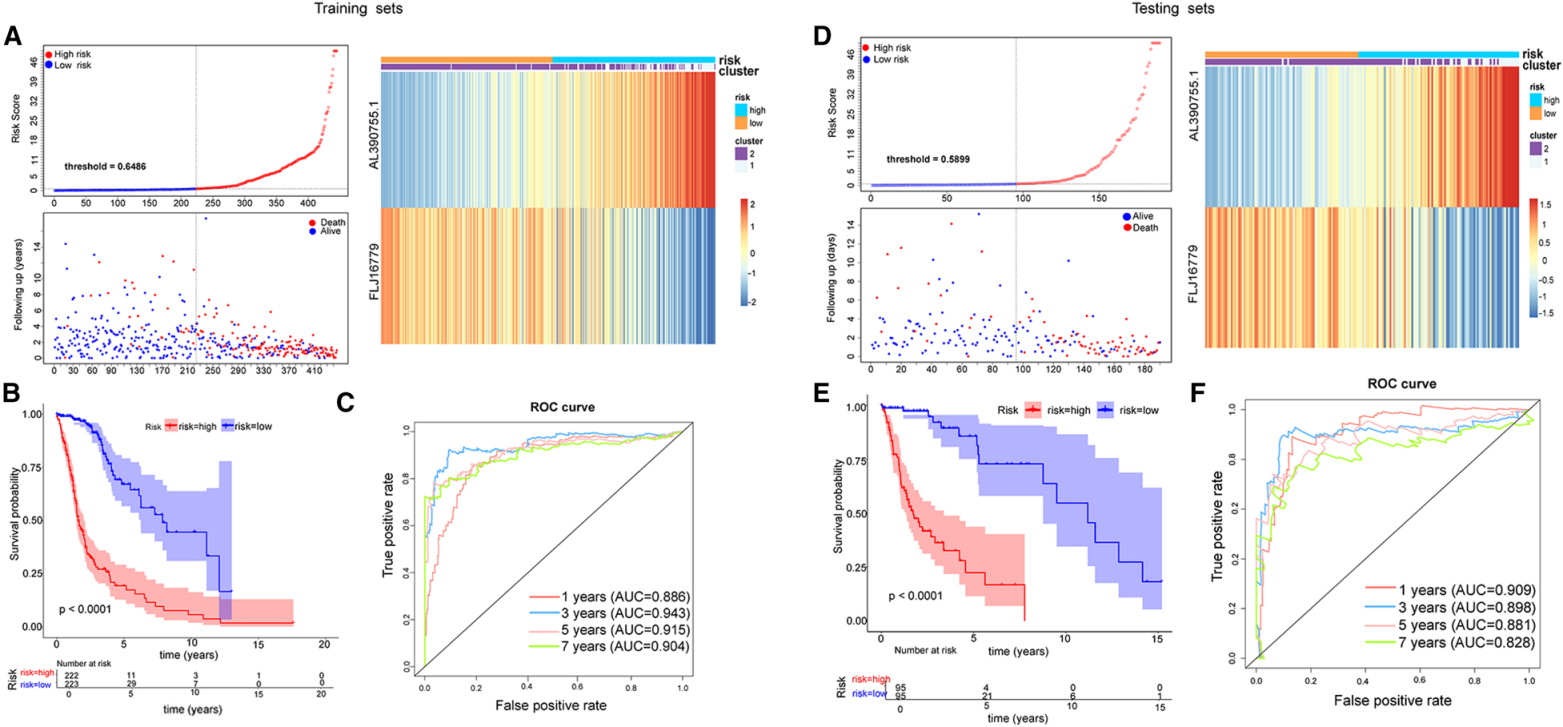
Construction of the glycolysis-related LncRNAs signature for survival prediction. **A**, **D** Risk score distribution, survival status for patients in low- and high-risk groups by the LncRNA signature in training and testing sets. **B**, **E** Kaplan–Meier curve based on the identified survival-related glycolysis LncRNAs in training and testing sets. **C**, **F** ROC curves of the signature for predicting1, 3, 5, 7- year survival of glioma in training and testing sets

Risk scores were calculated separately for each sample in the training and testing sets according to the aforementioned formula, and patients were categorized into high- and low-risk groups based on the median risk score of each set. In both the training and testing sets, with increasing risk scores, the number of patient deaths climbed sharply (Fig. 4A and D). Subsequently, K-M-survival analysis was performed on this prognostic feature, while AUC values were calculated by time-dependent ROC analysis for the accuracy of which in predicting survival, using the outcome variable. In the training set, K–M curves could effectively distinguish between high-risk and low-risk groups (Fig. 4B), and the risk scores all reached an AUC of 0.8 or more at 1 to 5 years (Fig. 4C). Similar results were also reproduced in the testing set (Fig. 4E and F). Based on the expression heatmap of the prognostic lncRNAs, which was observed in the high-risk group associated with poorer OS, *AL390755.1* was overexpressed. In contrast, *FLJ16779* was overexpressed in the low-risk group possessing a longer OS characteristic. Moreover, we found that the vast majority of Cluster 2 patients were in the low-risk group. These results suggested that the prognostic signature was highly sensitive and specific and that these signature lncRNAs could be utilized as prognostic biomarkers in clinically. Furthermore, the statistical tables of clinical information in the training and testing sets were displayed in Table 2.

**Table 2:** Clinical information of glioma patients in the training and testing sets

	Training sets			p	Testing sets			p
	Total (N = 417)	Expression			Total (N = 172)	Expression		
		High (N = 203)	Low (N = 214)			High (N = 88)	Low (N = 84)	
Gender				0.832				0.687
Female	184 (44.1%)	88 (43.3%)	96 (44.9%)		68 (39.5%)	33 (37.5%)	35 (41.7%)	
Male	233 (55.9%)	115 (56.7%)	118 (55.1%)		104 (60.5%)	55 (62.5%)	49 (58.3%)	
Age (years)				< 0.001				< 0.001
≥ 60	93 (22.3%)	74 (36.5%)	19 (8.9%)		42 (24.4%)	37 (42.0%)	5 (6.0%)	
< 60	324 (77.7%)	129 (63.5%)	195 (91.1%)		130 (75.6%)	51 (58.0%)	79 (94.0%)	
Cluster				< 0.001				< 0.001
1	86 (20.6%)	82 (40.4%)	4 (1.9%)		36 (20.9%)	34 (38.6%)	2 (2.4%)	
2	331 (79.4%)	121 (59.6%)	210 (98.1%)		136 (79.1%)	54 (61.4%)	82 (97.6%)	
Type				< 0.001				0.604
No PRT	85 (20.4%)	27 (13.3%)	58 (27.1%)		37 (21.5%)	16 (18.2%)	21 (25.0%)	
RT	52 (12.5%)	14 (6.9%)	38 (17.8%)		22 (12.8%)	12 (13.6%)	10 (11.9%)	
PT	38 (9.1%)	6 (3.0%)	32 (15.0%)		16 (9.3%)	7 (8.0%)	9 (10.7%)	
PT + RT	242 (58.0%)	156 (76.8%)	86 (40.2%)		97 (56.4%)	53 (60.2%)	44 (52. 4%)	
IDH				< 0.001				< 0.001
IDHmut-codel	114 (27.3%)	21 (10.3%)	93 (43.5%)		38 (22.1%)	7 (8.0%)	31 (36.9%)	
IDHmut-non-codel	158 (37.9%)	43 (21.2%)	115 (53.7%)		68 (39.5%)	16 (18.2%)	52 (61.9%)	
IDHwt	139 (33.3%)	134 (66.0%)	5 (2.3%)		63 (36.6%)	63 (71.6%)	0 (0%)	
Missing	6 (1.4%)	5 (2.5%)	1 (0.5%)		3 (1.7%)	2 (2.3%)	1 (1.2%)	
MGMT				< 0.001				0.001
+	300 (71.9%)	101 (49.8%)	199 (93.0%)		123 (71.5%)	46 (52.3%)	77 (91.7%)	
–	96 (23.0%)	81 (39.9%)	15 (7.0%)		40 (23.3%)	33 (37.5%)	7 (8.3%)	
Missing	21 (5.0%)	21 (10.3%)	0 (0%)		9 (5.2%)	9 (10.2%)	0 (0%)	
Grade				< 0.001				< 0.001
G2	136 (32.6%)	25 (12.3%)	111 (51.9%)		55 (32.0%)	16 (18.2%)	39 (46.4%)	
G3	148 (35.5%)	75 (36.9%)	73 (34.1%)		64 (37.2%)	29 (33.0%)	35 (41.7%)	
G4	92 (22.1%)	92 (45.3%)	0 (0%)		41 (23.8%)	39 (44.3%)	2 (2.4%)	
Missing	41 (9.8%)	11 (5.4%)	30 (14.0%)		12 (7%)	4 (4.5%)	8 (9.5%)	

*no PRT* no Pharmacotherapy and radiotherapy; *PT* Pharmacotherapy; *RT* radiotherapy; + methylated, − unmethylated

### Independent prognostic analysis

**Fig. 5:**
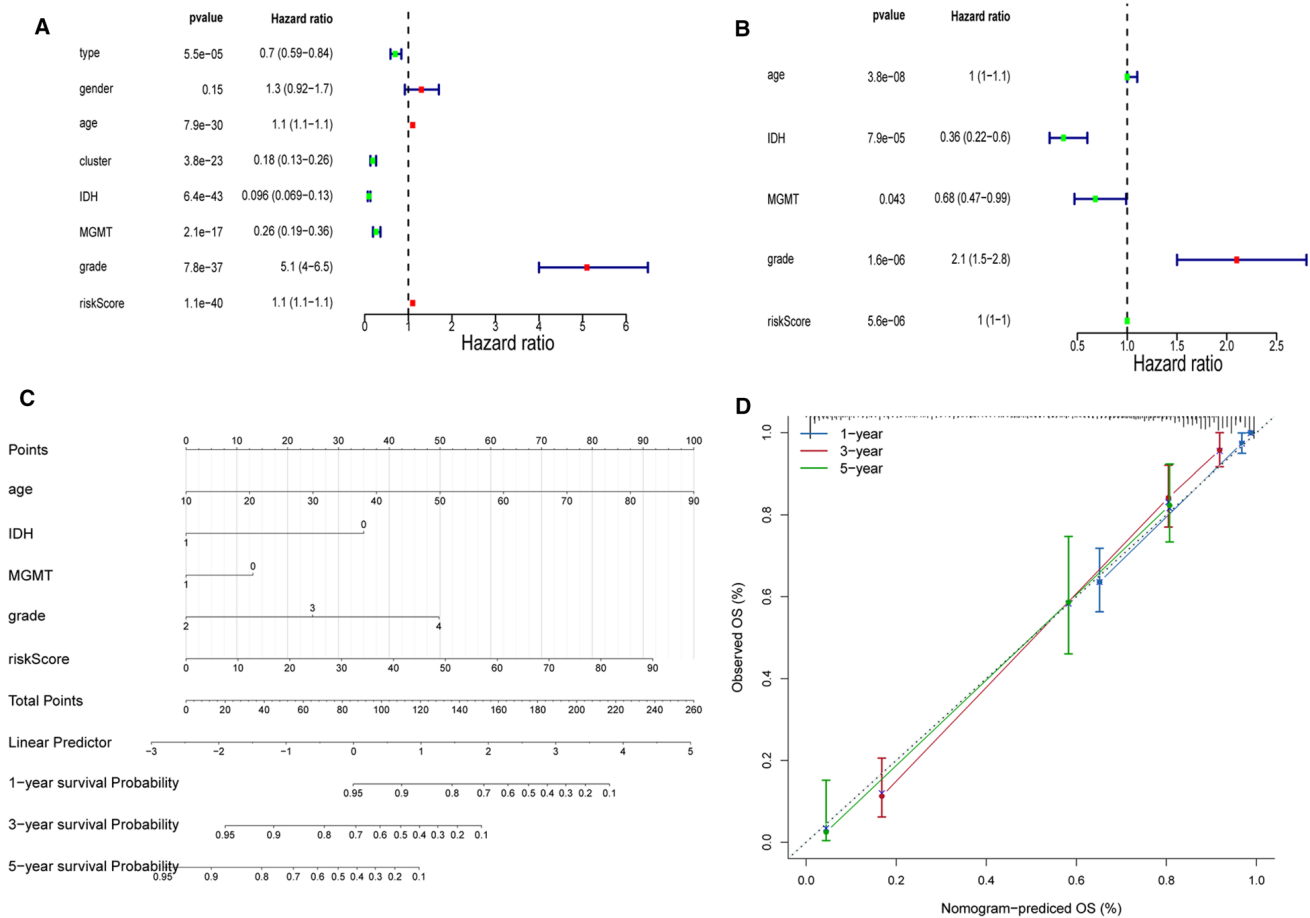
**A**, **B** Univariate (**A**) and multivariate (**B**) Cox analyses of clinicopathological characteristics and risk score. **C** Nomogram to predict the 1-, 3-, 5-year OS. **D** The calibration curve of the nomogram model prediction

Here, risk scores and clinicopathological characteristics (age, gender, treatment type, cluster, MGMT, grade, and IDH) of the full Gliomas sample in the TCGA database were included in the Cox analysis to explore their potential for independent prognosis. Ultimately, age, IDH, MGMT, grade, and risk score were identified as independent prognostic factors for glioma through univariate and multivariate Cox analyses (Fig. 5A and B). These independent prognostic factors were then incorporated into the Nomogram to explore the prediction of the Nomogram model for patient survival at 1, 3, and 5 years, which with a c-index of 0.8705766 (Fig. 5C). The status of each variable corresponded to a score, with a higher total score for a patient indicating poorer survival for that patient. Besides, the calibration curves also demonstrated that the Nomogram model was effective in predicting the survival of patients (Fig. 5D).

### Functional enrichment analysis of risk score-related DEGs

**Fig. 6:**
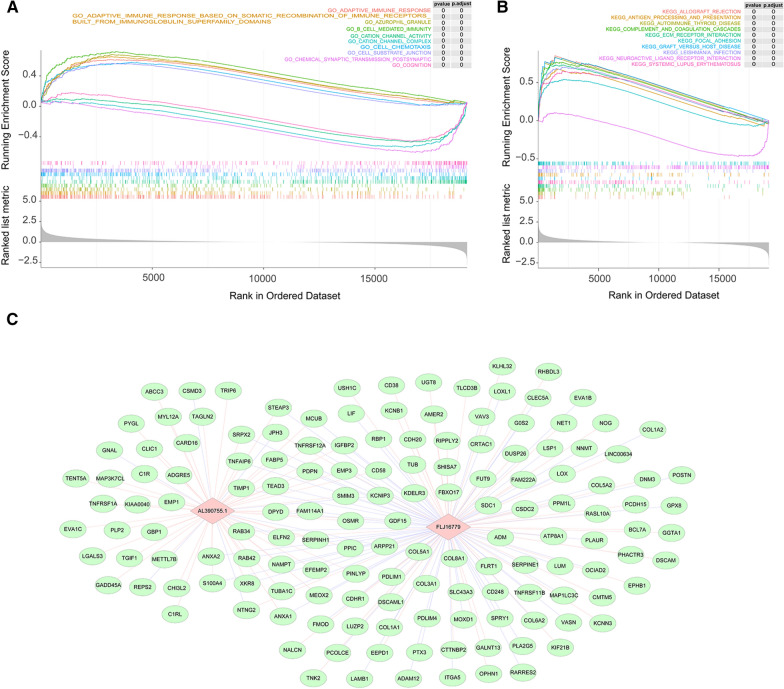
**A**, **B** Top10 GO (**A**) and KEGG (**B**) enrichment results by GSEA enrichment analysis. **C** The lncRNA-mRNA co-expression network by pearson correlation analysis. Two lncRNAs (pink rhombus) are connected to their respective co-expressed (|cor|≥ 0.6 and P < 0.05) mRNAs (blue circles) with lines (pink: postive correlation; purple: negative correlation)

To further reveal the potential function of prognostic lncRNAs, we first screened 680 risk score-related DEGs between high- and low-risk groups (Additional file 8: Table S8) and performed GSEA on them to reveal potential functions. Figure 6A and B illustrated the top10 GO and KEGG results, respectively. GO analysis revealed that these DEGs were mainly enriched in terms related to immune responses (‘ADAPTIVE IMMUNE RESPONSE’, ‘ADAPTIVE IMMUNE RESPONSE BASED ON SOMATIC RECOMBINATION OF IMMUNE RECEPTORS BUILT FROM IMMUNOGLOBULIN SUPERFAMILY DOMAINS’, ‘HUMORAL IMMUNE RESPONSE’, ‘NEGATIVE REGULATION OF IMMUNE SYSTEM PROCESS’, etc.) and immune cell regulation (‘LEUKOCYTE PROLIFERATION’, ‘REGULATION OF LYMPHOCYTE ACTIVATION’, ‘REGULATION OF T CELL ACTIVATION’, etc.). Notably, a multitude of biological processes associated with neurons (‘NEURON PROJECTION TERMINUS’, ‘NEURON TO NEURON SYNAPSE’, ‘REGULATION OF NEURON PROJECTION DEVELOPMENT’, etc.), neurotransmitters (‘NEUROTRANSMITTER RECEPTOR ACTIVITY’, ‘NEUROTRANSMITTER SECRETION’, ‘NEUROTRANSMITTER TRANSPORT’, etc.), and synapses (‘POSTSYNAPTIC MEMBRANE’, ‘POSTSYNAPTIC NEUROTRANSMITTER RECEPTOR ACTIVITY’, ‘POSTSYNAPTIC SPECIALIZATION MEMBRANE’, ‘PRESYNAPTIC MEMBRANE’, etc.) were significantly enriched. KEGG results indicated that risk score-related DEGs were significantly associated with immune disorders such as ‘AUTOIMMUNE THYROID DISEASE’ and ‘SYSTEMIC LUPUS ERYTHEMATOSUS’. Consistently, immune response-related pathways (‘CYTOKINE CYTOKINE RECEPTOR INTERACTION’, ‘PRIMARY IMMUNODEFICIENCY’, ‘NATURAL KILLER CELL MEDIATED CYTOTOXICITY’, etc.) were also significantly enriched. Moreover, the ‘AMINO SUGAR AND NUCLEOTIDE SUGAR METABOLISM’ pathway was inevitably featured in the results. More detailed results of GO and KEGG analysis were reported in Additional file 9: Table S9 and Additional file 10: Table S10.

Moreover, we illustrated the network of interactions of 150 risk score-related DEGs with prognostic lncRNAs for |cor|≥ 0.6 and *P* < 0.05 in Fig. 6C. Among them, 35 genes were common DEGs for both prognostic lncRNAs (Table 3). Elaborately, *ARPP21*, *JPH3*, *NTNG2*, *KCNIP3*, and *ELFN2* were positively correlated with lncRNAs *FLJ16779*, but negatively associated with *AL390755.1*. The remaining 30 genes such as *EMP3*, *TNFRSF12A*, *MCUB*, and *ANXA2* were positively associated with lncRNA *AL390755.1*, but negatively related to *FLJ16779*.

**Table 3:** List of common risk score-related DEGs for prognostic lncRNAs (|cor|≥ 0.6 and *P* < 0.05)

Gene name	Correlation	
	FLJ16779	AL390755.1
ELFN2	0.696	− 0.623
KCNIP3	0.677	− 0.626
NTNG2	0.637	− 0.645
JPH3	0.625	− 0.614
ARPP21	0.614	− 0.614
EMP3	− 0.669	0.700
TNFRSF12A	− 0.615	0.684
MCUB	− 0.697	0.679
ANXA2	− 0.630	0.670
ANXA1	− 0.616	0.666
CD58	− 0.636	0.663
RAB42	− 0.626	0.600
FABP5	− 0.633	0.601
MEOX2	− 0.620	0.602
XKR8	− 0.600	0.605
EFEMP2	− 0.626	0.606
DPYD	− 0.676	0.606
NAMPT	− 0.632	0.610
PPIC	− 0.630	0.610
TEAD3	− 0.608	0.616
FAM114A1	− 0.622	0.620
PINLYP	− 0.636	0.620
SERPINH1	− 0.609	0.621
RAB34	− 0.669	0.623
OSMR	− 0.620	0.628
SRPX2	− 0.652	0.628
GDF15	− 0.671	0.630
TNFAIP6	− 0.650	0.631
S100A4	− 0.634	0.631
STEAP3	− 0.624	0.643
TUBA1C	− 0.693	0.647
TIMP1	− 0.694	0.651
PDPN	− 0.638	0.655
SMIM3	− 0.668	0.656
IGFBP2	− 0.665	0.657

### The impact of risk scores on the immune landscape of glioma patients

**Fig. 7:**
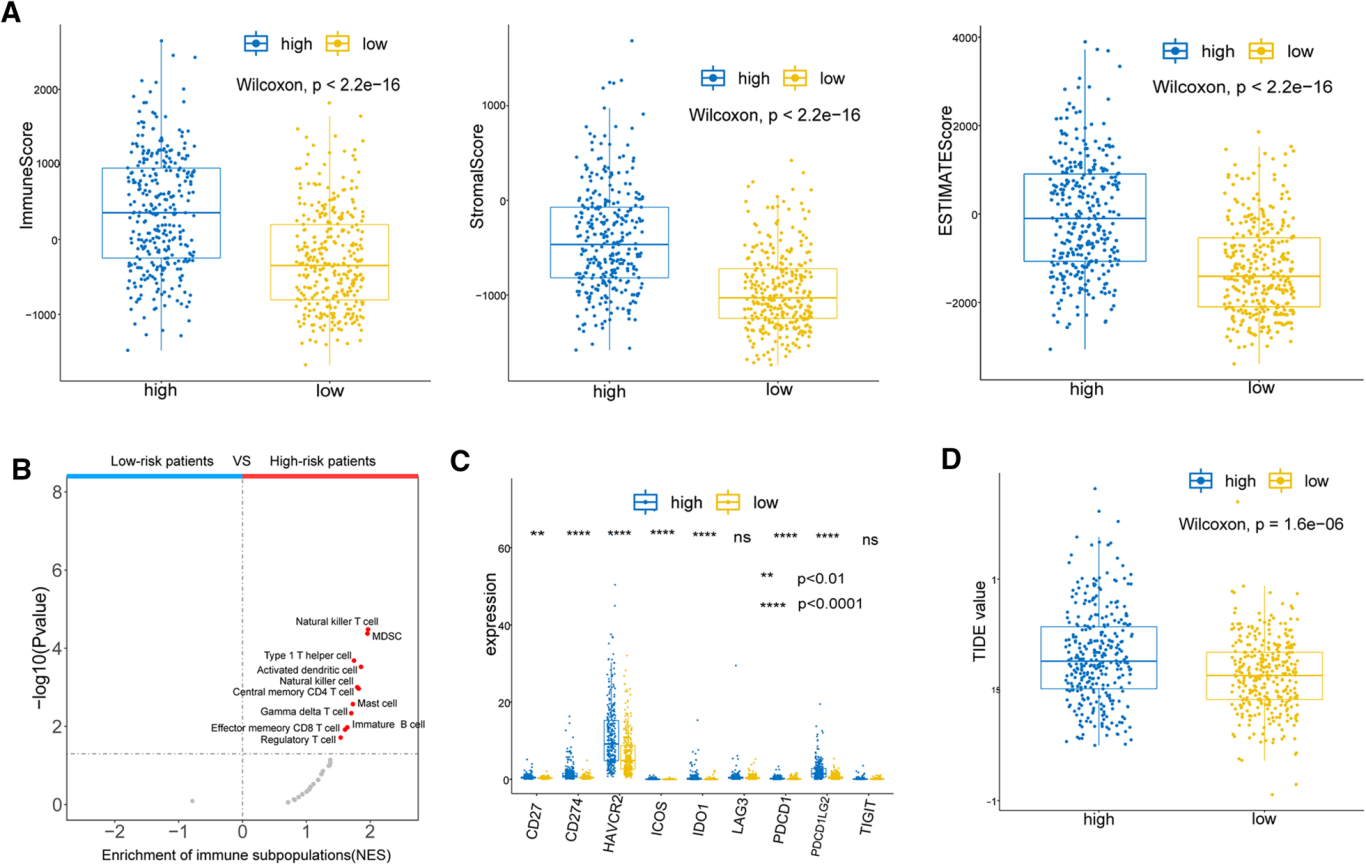
**A** Comparison of immune, stromal, and ESTIMATE scores between the high-risk group and low-risk group. **B** Volcano plots for the enrichment of immune cell types based on the normalized NES score between low-risk and high-risk patients from the GSEA. **C** The relationship between risk score and the expression of nine ICIs. **D** Comparison of TIDE scores between the high-risk group and the low-risk group

Inspired by the results of the ssGSEA, we hypothesized that prognostic lncRNAs may operate in the patient’s immune microenvironment to influence patient outcomes. ESTIMATE analysis indicated that the immune, stromal, and ESTIMATE scores were significantly higher in the high-risk group than in the low-risk group, suggesting that the high-risk group had more components of the immune microenvironment (Fig. 7A). Subsequent immune cell enrichment analysis demonstrated that more immune cells, such as Natural killer T cells, Myeloid-derived suppressor cell (MDSC), and Type 1T helper cell, were presented in the high-risk group (Fig. 7B). This evidence suggested that prognostic lncRNAs were involved in the altered immune microenvironment of patients.

### The relationship between risk scores and patient response to ICI therapy

Over the past decade, ICIs have proven to be promising agents for many solid tumor malignancies, adapting this treatment strategy to an increasingly important role in Neuro-oncology [1, 34]. Therefore, we investigated the potential relationship between risk score and the expression of nine ICIs. Seven ICIs, excluded LAG3 and TIGIT were detected to have higher expression levels in the high-risk group, which may be related to the higher tumor grade of patients in which group (Fig. 7C). The TIDE algorithm then predicted the sensitivity of patients in the high- and low-risk groups to ICI treatment. TIDE scores were significantly higher in the high-risk group than in the low-risk group, suggesting that patients in the low-risk group may benefit from ICI treatment (Fig. 7D).

### Expression of AL390755.1 and FLJ16779 mRNA in NHA, U87 and A172 cells

**Fig. 8:**
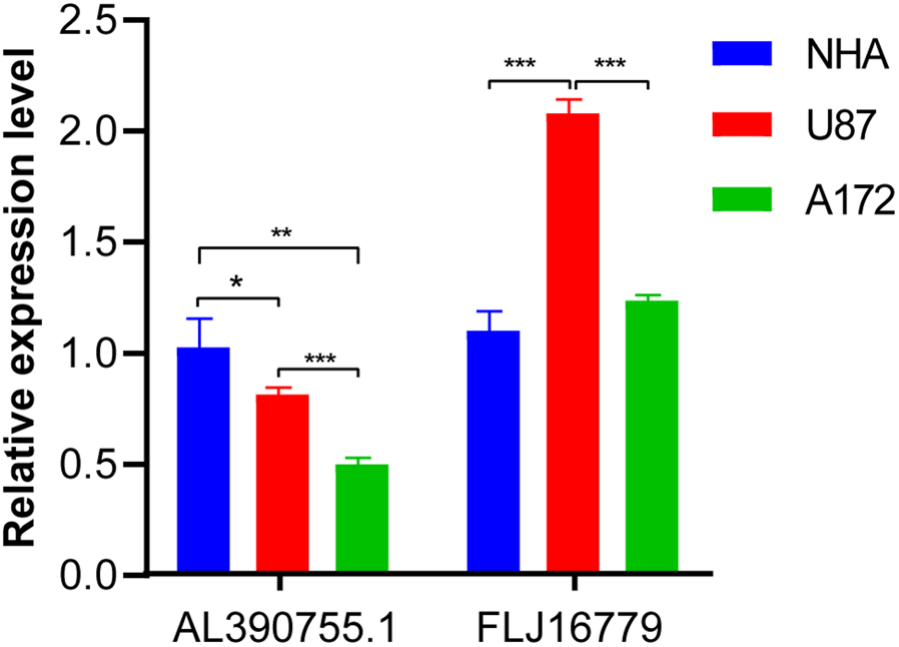
Expression of AL390755.1 and FLJ16779 in NHA, U87 and A172 Cells, ^∗^*p* < 0*.*5, ^∗∗^*p* < 0*.01,* ∗∗∗*p* < 0*.*001

By qRT-PCR validation of the expression of the two lncRNAs, we found that the expression of *AL390755.1* was significantly lower in glioma cells than that in NHA cells, while the expression of *FLJ16779* showed the opposite results (Fig. 8).

## Discussion

Glioma is one of the most common, aggressive and challenging malignant tumors in neurosurgery. In this study, we identified an efficient prognostic model consisting of two glycolysis-related lncRNAs using TCGA databases. This model demonstrated strong predictive capacity for glioma patient prognosis as patients in the high-risk group had poorer overall survival compared to those in the low-risk group. Additionally, the nomogram model showed superior performance in predicting prognosis. GO analysis revealed that DEGs associated with risk scores were mainly involved in immune responses and immune cell regulation. KEGG results indicated that risk score-related DEGs were significantly associated with immune disorders and immune response-related pathways. Immune landscape analysis revealed extreme enrichment of immune cells in the high-risk group. Furthermore, patients in the low-risk group appeared to benefit more from ICI treatment. These findings suggest that glycolysis-related lncRNAs could serve as important biomarkers and potenial therapeutic targets for glioma.

Recently, the fields of tumor metabolism and immune oncology have received significant attention. Gliomas cells display higher levels of glycolytic activities compared with normal brain tissue, particularly in glioblastomas (GBM) [5], which are widely acknowledged to be a hallmark of immune cell activation [3]. Zehang Jiang et al. found that there is a positive correlation between glycolytic activity and immune score across all 14 cancer types, with GBM and LGG having the highest correlation [14]. Previous cancer imaging studies have revealed the complex relationship between tumor glucose metabolism rate and immune microenvironment [9, 19]. Reversing this metabolic program could provide adequate glucose for immune cells to stimulate antitumor response. Although checkpoint inhibitors and CAR-T cell therapy are currently the most common immunotherapies for cancer patients, only 20–40% of patients respond to immunotherapy [12, 22], the impact of glucose metabolism shift on immune cell function may be another explanation for this issue. Modulating glioma metabolism represents a logical therapeutic approach. Our study showed that these glycolysis-associated lncRNAs were mainly involved in immune responses based on functional enrichment analysis. Additionally, immune landscape analysis suggested an extreme enrichment of immune cells in the high-risk group, patients in the low-risk group were more likely to benefit from ICI treatment. This observation is consistent with recent findings that increased tumor glycolysis suppressed antitumor immunity [4], and immunotherapy was more favorable in tumors with low glycolysis [17, 27, 31]. Therefore, adjusting cell metabolism along with immunotherapy might be an effective treatment regime. However, large cohorts are required to verify the association between tumor glycolysis and immunotherapy response.

Several studies have indicated that specific metabolism-related genes may serve as prognostic indicators for patients with glioma [6, 10, 25, 26, 32]. In contrast to our results, Wang Jia and colleagues identified six glycolysis-related lncRNAs in glioma patients based on the CGCA database, the best results they obtained had ROC-AUC 0.875 and 0.879 for the training and validation set, respectively [25, 26]. Other models displayed best ROC-AUC ranging from 0.847 to 0.873 [10, 32]. In contrast, our prediction model achieved ROC-AUC 0.943 and 0.909 for the training and validation set, respectively, while the corrected C-index was 0.8705766, suggesting superior accuracy in predicting patient outcomes. Our identification of these two glycolysis-related lncRNAs signatures has substantial clinical significance due to their high sensitivity and specificity as prognostic biomarkers.

Specifically, we have reported a significant finding that *FLJ16779* and *AL390755.1* were confirmed for the first time to be correlated with the prognosis prediction of glioma. The study of Yongqiang Wang et al. complements these findings by establishing a robust three-lncRNA model for predicting the OS of gastric cancer patients, including *OVAAL*, *FLJ16779* and *FAM230D* [25, 26], suggesting the crucial role of *FLJ16779* in predicting the prognosis of various types of cancers. Little information is available about these glycolysis-related lncRNAs in glioma and other tumors*,* which prompted us to evaluate the potential function by using GSEA. GO and KEGG analysis revealed that these DEGs were mainly enriched in terms related to immune responses and immune response-related pathways. Consequently, we postulate that the interaction of these two glycolysis-related genes with immune system responses may play a critical role in the carcinogenesis and progression of glioma. Thus, further experimental trials are warranted to validate these preliminary results and address any outstanding questions.

In this study, we have identified a two glycolysis-related lncRNA signature for predicting the prognosis of glioma patients based on TCGA database. This finding provides a promising avenue for exploring new immune checkpoint inhibitor (ICI) therapeutic targets for glioma patients. However, there were certain limitations in this study. It was only a preliminary study exploring the relationship between glycolysis level and immune cell infiltration, and the exact mechanism of action and regulatory relationship need to be further studied.

## Materials and methods

### Data source

A total of 675 samples from The Cancer Genome Atlas (TCGA)-lower grade gliomas (LGG) and -Glioblastoma (GBM) databases were utilized in this study, of which 5 were normal samples and 670 were glioma samples. Of the glioma samples, 635 samples had survival information and 580 samples had complete clinical information. Glycolysis genes (Additional file 1: Table S1) were downloaded from the Molecular Signatures Database (MSigDB) for all genes in the glycolytic pathway, including BIOCARTA GLYCOLYSIS PATHWAY, GO GLYCOLYTIC PROCESS, HALLMARK GLYCOLYSIS, KEGG GLYCOLYSIS GLUCONEOGENESIS, and REACTOME GLYCOLYSIS [28].

### Consistent clustering

The consensus clustering was accomplished in R by using the ConsensusClusterPlus package. Selection of glioma subtypes based on the expression profile of the glycolysis genes employing a k-means clustering approach. The optimal number of clusters was decided by the cumulative distribution function (CDF) curve of the consensus scores and the consensus matrix heatmap. The Kaplan–Meier (K–M) curve was adopted to evaluate the prognosis of the different subtypes and *P* < 0.05 was considered significant.

### Pathway analysis of different subtypes

Kyoto Encyclopedia of Genes and Genomes (KEGG) pathway identification was performed by single-sample gene set enrichment analysis (ssGSEA) to assess pathway variations between subtypes. Briefly, ssGSEA calculated an enrichment score (ES) for each KEGG pathway in each sample within each cluster via the GSVA software package. Each ssGSEA ES represented the extent to which the KEGG pathway was up- or down-regulated in the sample (Additional file 2: Table S2).

### Differential analysis

The differential expression analysis would be performed in R using the limma package to assess the expression distribution of lncRNAs in different subtypes. The lncRNAs satisfying |log_2_ fold change (FC)|> 2 and *P* < 0.05 were considered as DE-lncRNAs and included in the subsequent analysis. Furthermore, genes satisfying |log_2_ FC|> 1 and *P* < 0.05 were considered to be the risk score-related DEGs between the high- and low-risk groups.

### Construction and assessment of the prognostic feature

Here, we worked with a sample of 635 TCGA-glioma subjects with survival information. First, we randomly divided the sample of 635 cases into a training set (n = 445) and an internal validation set (n = 190) in a ratio of 7:3. The training set was used to locate prognostic lncRNAs and the assessment of the predictive validity of the prognostic feature, and the internal validation set was only designed to validate the predictive validity of the prognostic feature. Subsequently, DE-lncRNAs screened by univariate Cox regression analysis (*P* < 0.05) were further included in multivariate Cox regression analysis with a step function to determine the best variables to use to construct a prognostic feature. The risk score for each patient in the training and internal validation sets was calculated based on the coefficient (coef) value (Additional file 3: Table S3) and expression of each prognostic lncRNA as shown in the formula below:

$${\text{Risk score}} = \frac{e^{{\text{sum(each gene's expression levels}} \times {\text{corresponding coefficient}}})}{e^{{\text{sum (each gene's mean expression levels}}\,\times {\text{corresponding coefficient)}}}}$$

Patients were categorized into high- and low-risk groups based on the median risk score. The K-M analysis for detecting the difference in OS between the high- and low-risk groups was used. To assess and validate the prognostic predictive validity of the prognostic feature, ROC curves were performed in both the training set and the internal validation set.

### Creation of the Nomogram

Univariate and multivariate Cox analyses were conducted to explore the independent prognostic factors in patients with glioma. Variables initially included were risk score, age, gender, type of treatment (type), IDH mutation type (IDH), grade, MGMT status (MGMT), and sub-clustering (cluster). Ultimately, independent prognostic factors identified by multivariate Cox analysis (*P* < 0.05) would be available for the construction of the Nomogram. Additionally, calibration curves were obtained to assess the predictive accuracy of the Nomogram model for patients with 1, 3, and 5-year OS.

### GSEA in risk score-related DEGs

Gene Ontology (GO) and KEGG analysis of risk score-related DEGs was implemented based on R software using GSEA in the clusterProfiler package. Here, as functional enrichment studies of lncRNAs are not yet straightforward, we focused on potential functions with risk score-related DEGs to understand the biological processes and pathways that lncRNAs may be involved in. GO analysis was used to reveal the potential biological functions of risk score-related DEGs, while KEGG was responsible for the enrichment of the pathways.

### lncRNA-mRNA network

Due to the large size of risk score-related DEGs, we filtered out risk score-related DEGs that were strongly correlated with prognostic lncRNAs (|coefficient (cor)|≥ 0.6 and *P* < 0.05) using Pearson correlation analysis (Additional file 4: Table S4 and Additional file 5: Table S5). The lncRNA-mRNA network was then mapped and embellished using Cytoscape software.

### Immune landscape analysis

The stromal score, immune score, and ESTIMATE score for each sample (n = 635) were calculated by applying the estimate R package. Further Wilcoxon tests were performed to assess the relationship between these scores and risk score (n_high-risk_ = 317, n_low-risk_ = 318).

In addition, we performed a GSEA using the clusterProfiler package to identify immune cell types that differed between the high- and low-risk groups. Briefly, genes between the high- and low-risk groups were sorted by |log_2_ FC| and then GSEA of immune cells was performed. The normalized ES (NES) was used to identify differences in immune cell types between the two groups.

### Cell culture and qRT-PCR

NHA were purchased from Lonza and cultured using an AGM Bullet Kit™ (Lonza, Walkersville, MD) as recommended by the manufacturer. U87 and A172 cells were purchased from ATCC and were cultured in Dulbecco’s modified.

Eagle’s medium (Gibco, Carlsbad, CA) containing 10% fetal bovine serum.

(Gibco, Carlsbad, CA) according to standard protocols. Then, place the cells in a 37 °C, 5% CO2 incubator for culture. Change the medium once a day. The RNA is extracted when the cells grow to 80% confluent. Use TRIzol (ThermoFisher Scientific, USA) to extract total cell RNA. Follow the steps of PrimeScrip reverse transcription kit (Takara, Japan) to reverse transcription into cDNA. Configure the PCR reaction system and analyze it according to the SYBR Premix Ex Taq (Takara, Japan) instruction. Human actin was used as an endogenous control, the relative gene expression was calculated by the 2 ^−ΔΔCt^ method. Primer sequence utilized for the qRT-PCR analysis is listed in Table 4. Repeat the experiment 3 times independently for each sample.

**Table 4:** qRT-PCR primer sequence

Primer sequences (5′–3′)		
AL390755.1	Forward:	ACCCATCTTATCCAGGGGCT
	Reverse:	CCTCACATCGCTGTCCCTTT
FLJ16779	Forward:	TTTAGTGCCTAGCAGCAGCC
	Reverse:	CCACAGCCCTAACCTGTACG
Human actin	Forward:	GACAGGATGCAGAAGGAGATTACT
	Reverse:	TGATCCACATCTGCTGGAAGGT

### Statistical analysis

Survival analysis was performed in the survival package. The ggplot2 package was used to plot volcanoes. pROC was used for ROC curve generation. Nomograms were obtained using the rms package. Sensitivity prediction for ICI treatment was achieved through the TIDE online website (http://tide.dfci.harvard.edu/). R software was employed for statistical analysis and *P* < 0.05 was considered statistically significant.

## Supplementary Information

**Additional file 1.** Glycolysis genes download from the Molecular Signatures.

**Additional file 2.** Enrichment score for each KEGG pathway in each sample within each cluster.

**Additional file 3.** Differentially expressed lncRNAs screened by multivariate Cox regression analysis.

**Additional file 4.** Genes strongly correlated with AL390755.1 using Pearson correlation analysis. (|coefficient (cor)| ≥ 0.6 and P < 0.05).

**Additional file 5.** Genes strongly correlated with FLJ16779 using Pearson correlation analysis. (|coefficient (cor)| ≥ 0.6 and P < 0.05)

**Additional file 6.** Pathway enrichment scores between the two clusters.

**Additional file7.** Differentially expressed glycolysis-related lncRNAs between Cluster1 and Cluster2.( |log2 (fold change)| > 2 and P_value < 0.05).

**Additional file 8.** Risk score-related differentially expressed genes between high- and low-risk groups.

**Additional file 9. ** GO results of risk score-related differentially expressed genes.

**Additional file 10.** KEGG results of risk score-related differentially expressed genes.

## Data Availability

Our study is based on public databases, the patients involved in the database have obtained ethical approval. Users can download relevant data for free for research and publish relevant articles.

## References

[1] Almquist DR, Ahn DH, Bekaii-Saab TS (2020). The role of immune checkpoint inhibitors in colorectal adenocarcinoma. BioDrugs.

[2] Bai Y, Lin H, Chen J, Wu Y, Yu S (2021). Identification of prognostic glycolysis-related lncRNA signature in tumor immune microenvironment of hepatocellular carcinoma. Front Mol Biosci..

[3] Buck MD, Sowell RT, Kaech SM, Pearce EL (2017). Metabolic instruction of immunity. Cell.

[4] Cascone T, McKenzie JA, Mbofung RM, Punt S, Wang Z, Xu C (2018). Increased tumor glycolysis characterizes immune resistance to adoptive T cell therapy. Cell Metab..

[5] Charles NA, Holland EC, Gilbertson R, Glass R, Kettenmann H (2011). The brain tumor microenvironment. Glia.

[6] Chen C, Shi Y, Li Y, He ZC, Zhou K, Zhang XN (2017). A glycolysis-based ten-gene signature correlates with the clinical outcome, molecular subtype and IDH1 mutation in glioblastoma. J Genet Genomics.

[7] Fan L, Huang C, Li J, Gao T, Lin Z, Yao T (2018). Long noncoding RNA urothelial cancer associated 1 regulates radioresistance via the hexokinase 2/glycolytic pathway in cervical cancer. Int J Mol Med.

[8] Ganapathy-Kanniappan S, Geschwind JF (2013). Tumor glycolysis as a target for cancer therapy: progress and prospects. Mol Cancer.

[9] Haghighat Jahromi A, Barkauskas DA, Zabel M, Goodman AM, Frampton G, Nikanjam M (2020). Relationship between tumor mutational burden and maximum standardized uptake value in 2-[(18)F]FDG PET (positron emission tomography) scan in cancer patients. EJNMMI Res.

[10] He Z, Wang C, Xue H, Zhao R, Li G (2020). Identification of a metabolism-related risk signature associated with clinical prognosis in glioblastoma using integrated bioinformatic analysis. Front Oncol.

[11] Huang P, Zhu S, Liang X, Zhang Q, Luo X, Liu C (2021). Regulatory mechanisms of LncRNAs in cancer glycolysis: facts and perspectives. Cancer Manag Res.

[12] Innao V, Allegra AG, Musolino C, Allegra A (2020). New frontiers about the role of human microbiota in immunotherapy: the immune checkpoint inhibitors and CAR T-cell therapy era. Int J Mol Sci.

[13] Jiang Y, Chen J, Ling J, Zhu X, Jiang P, Tang X (2021). Construction of a glycolysis-related long noncoding RNA signature for predicting survival in endometrial cancer. J Cancer.

[14] Jiang Z, Liu Z, Li M, Chen C, Wang X (2019). Increased glycolysis correlates with elevated immune activity in tumor immune microenvironment. EBioMedicine.

[15] Kesarwani P, Kant S, Prabhu A, Chinnaiyan P (2017). The interplay between metabolic remodeling and immune regulation in glioblastoma. Neuro Oncol.

[16] Liu Y, He D, Xiao M, Zhu Y, Zhou J, Cao K (2021). Long noncoding RNA LINC00518 induces radioresistance by regulating glycolysis through an miR-33a-3p/HIF-1alpha negative feedback loop in melanoma. Cell Death Dis.

[17] Madden MZ, Rathmell JC (2021). The complex integration of T-cell metabolism and immunotherapy. Cancer Discov.

[18] Ostrom QT, Patil N, Cioffi G, Waite K, Kruchko C, Barnholtz-Sloan JS (2020). CBTRUS statistical report: primary brain and other central nervous system tumors diagnosed in the United States in 2013–2017. Neuro Oncol.

[19] Park C, Na KJ, Choi H, Ock CY, Ha S, Kim M (2020). Tumor immune profiles noninvasively estimated by FDG PET with deep learning correlate with immunotherapy response in lung adenocarcinoma. Theranostics.

[20] Pavlova NN, Thompson CB (2016). The emerging hallmarks of cancer metabolism. Cell Metab.

[21] Poteet E, Choudhury GR, Winters A, Li W, Ryou MG, Liu R (2013). Reversing the Warburg effect as a treatment for glioblastoma. J Biol Chem.

[22] Sharma P, Hu-Lieskovan S, Wargo JA, Ribas A (2017). Primary, adaptive, and acquired resistance to cancer immunotherapy. Cell.

[23] Vander Heiden MG (2011). Targeting cancer metabolism: a therapeutic window opens. Nat Rev Drug Discov.

[24] Velpula KK, Bhasin A, Asuthkar S, Tsung AJ (2013). Combined targeting of PDK1 and EGFR triggers regression of glioblastoma by reversing the Warburg effect. Cancer Res.

[25] Wang Y, Zhang H, Wang J (2020). Discovery of a novel three-long non-coding RNA signature for predicting the prognosis of patients with gastric cancer. J Gastrointest Oncol.

[26] Wang Y, Zhou W, Ma S, Guan X, Zhang D, Peng J (2020). Identification of a glycolysis-related LncRNA signature to predict survival in diffuse glioma patients. Front Oncol..

[27] Watson MJ, Vignali PDA, Mullett SJ, Overacre-Delgoffe AE, Peralta RM, Grebinoski S (2021). Metabolic support of tumour-infiltrating regulatory T cells by lactic acid. Nature.

[28] Wu C, Cai X, Yan J, Deng A, Cao Y, Zhu X (2020). Identification of novel glycolysis-related gene signatures associated with prognosis of patients with clear cell renal cell carcinoma based on TCGA. Front Genet..

[29] Xiao ZD, Zhuang L, Gan B (2016). Long non-coding RNAs in cancer metabolism. BioEssays.

[30] Xu Z, Zhang D, Zhang Z, Luo W, Shi R, Yao J (2020). MicroRNA-505, suppressed by oncogenic long Non-coding RNA LINC01448, acts as a novel suppressor of glycolysis and tumor progression through inhibiting HK2 expression in pancreatic cancer. Front Cell Dev Biol..

[31] Zappasodi R, Serganova I, Cohen IJ, Maeda M, Shindo M, Senbabaoglu Y (2021). CTLA-4 blockade drives loss of Treg stability in glycolysis-low tumours. Nature.

[32] Zhang C, Wang M, Ji F, Peng Y, Wang B, Zhao J (2021). A novel glucose metabolism-related gene signature for overall survival prediction in patients with glioblastoma. Biomed Res Int.

[33] Zhang Z, Fang E, Rong Y, Han H, Gong Q, Xiao Y (2021). Hypoxia-induced lncRNA CASC9 enhances glycolysis and the epithelial–mesenchymal transition of pancreatic cancer by a positive feedback loop with AKT/HIF-1alpha signaling. Am J Cancer Res.

[34] Zhao J, Chen AX, Gartrell RD, Silverman AM, Aparicio L, Chu T (2019). Immune and genomic correlates of response to anti-PD-1 immunotherapy in glioblastoma. Nat Med.

